# Philadelphia Hospital

**Published:** 1838-01-17

**Authors:** 


### PHILADELPHIA HOSPITAL.

Saturday, 30th December.—Dr. Gibson presented to the class the negro operated upon 16th December, in whom he had removed the greater portion of the upper jaw. Upon examination of the tumour which I removed, said he, I found it to be an osteo-sarcoma. This is a mixture of bony and fleshy matter, in which the bone is generally so arranged, as to be divided into a collection of cells, in which the fleshy matter is deposited. The matter in these cells may vary in size from a hazel nut to a pea; I have sometimes seen it as large as a pullet's egg. At the commencement of my career as a surgeon, I was in the habit of performing bold and decided operations for the removal of osteo-sarcomatous tumours. Invariably, however, I found them to return. They seemed to approach in their nature to cancer or fungus hœmatodes, more particularly the former, and I met with no success in my efforts to extirpate them. If I had known the tumour in case, to be an osteo-sarcoma, I would not have meddled with it. This I could not determine, until after I cut into it.

The condition of the patient is however decidedly improved since the operation. The deep seated, racking pain, which tormented him in the jaw, extending up into the head, has entirely left him.—For two or three days after the operation, I kept him on a low diet. A vast quantity of foul and fœtid matter formed; and the man was salivated. The slough separated in 4 or 5 days, and granulations were thrown out. The smoothness of the surface of the wound was interrupted by spicula of bone, several of which were removed from the left side by Dr. Lajus. It is impossible to prevent the occurrence of exfoliations of this sort, after the bruising to which the bone is liable from the use of the mallet and gouge. The spicula, in this instance, I am inclined to think, are the remains of the disease, the jaw having still a disposition to throw out this sort of diseased matter.

The patient is in the enjoyment of much better health, since the removal of the tumour. He has had no fever, except on the day immediately succeeding the operation. The surface of the wound is covered with healthy granulations of a rose color. Whether this improvement be of a permanent character or not, time only can show.

Surgeons differ in opinion, as to the possibility of curing osteo-sarcoma. Perhaps this difference of opinion may arise from the fact, that bony tumours of various characters are apt to be confounded together. Spina ventosa has been mistaken for osteo-sarcoma, although the former is a mere conversion, of the periosteum into a shell of bony matter. So likewise has exostosis, which is a tumour on the surface of a bone, arising from superabundance of bony matter, and ordinarily free from malignancy. Necrosis is a disease of a different nature. This is the death of the old bone, and the reproduction of a new, which incloses the former. The interior becomes dead, from disease in the periosteum, which forms a shell around it, through which holes are left, for the discharge of sequestra or fragments of the old bony matter. The cure of this disease is by a long and tedious process, the operation of nature, which loosens the old bone and discharges it externally. It is best to suffer nature to take her own course; for no interference of surgical art will prove of any avail.

Dr. Gibson here called the attention of the class to the instruments with which he had operated on the previous Saturday. They are, said he, a set which I contrived, some years since, for the removal of a fungus in the antrum. You observed that the knife, which I used, seemed to make its way, with great facility, though in reality, I was obliged to use a considerable degree of force. It was besides no ordinary scalpel, but tempered like a sword, remarkably thick, and would cut iron itself. It was made expressly to cut bone, and with it, I was able to produce the same effect, as by striking with a chisel or gouge. Here are several knives of the kind, of different shapes, some curved, some angular, some like a shoemaker's pegging knife; they are all well calculated to cut through bone. This saw I thought I might have occasion to use; but the bone was so soft, that there was no necessity for it. The saw has this advantage, that you may apply it in different directions, cutting perpendicularly, horizontally or obliquely, as the case may require. I show you the diseased mass which I extirpated. You observe the fleshy matter springing from the bony cells; by passing the spatula into them, I can feel these breaking up. When the fleshy matter is removed by maceration, or other means, the preparation resembles a piece of coral, or a dried cedar bush.

I wish you to bear in mind, gentlemen, that, in this operation, I pushed the use of the knife to the farthest extent, that was admissible. I might have penetrated into the antrum, or cut away the whole of both upper jaw bones; but I know of no advantage to have been anticipated from such a course. If hereafter I detect any return of the disease, I can follow up what I have now done, with the saw. A central portion of the palate bone, has, you see, been left to support the bones of the nose, the shape of which would otherwise have been sunk.

If you examine the man's mouth, you will notice the white, granulated appearance, which the whole surface of the wound presents; and a very healthy appearance it is, although it is but a fortnight since the operation. Observe too how nicely the parts have been smoothed off and rounded by the granulating process. The general health of the patient is, as I have already stated, rapidly improving. The entire absence of hœmorrhage, notwithstanding the number of vessels wounded, has been a most fortunate circumstance.

I took an opportunity, two or three weeks since, gentlemen, to bring under your notice some interesting examples of diseases of the joints. To day I propose to call your attention to some cases of an analogous nature, exemplifying the subject of diseases of the spine, and to point out a very important distinction which exists between affections of this character, although they are apt to be confounded both by writers and practitioners. Many look upon all diseases of the spine as essentially the same, and trace them to the same exciting cause. In reality, they differ widely in their origin, and require very opposite plans of treatment.

Young boys and girls not unfrequently suffer from distortion of the spine, and an unnatural twist in their backs, giving rise to considerable deformity: this is occasioned by immoderately rapid growth, and is not disease. When a lad shoots up very quickly, as the bony matter is developed in excessive quantity, it becomes softer in its character; the ligaments too give way in a degree, and the natural consequence is, a twist to the right or the left, or anteriorly or posteriorly. To remedy this, you must pursue a very different mode of treatment from that which is proper for the management of caries of the spine. The one requires exercise, the other, absolute rest: unfortunately, they are but too generally treated alike. It is my intention first to discuss the subject of caries of the spine, and afterwards to take up that of simple softening of the bony structure of the vertebræ.

This hospital presents numerous cases of caries of the spine, in almost every stage of the disease. Many of them are completely cured; but, in every instance, this has occurred through the medium of anchylosis. The deformity consequent upon this process can, of course, never be removed, as it has been the very essence of the cure itself.

Caries of the spine occurs almost invariably in individuals of a scrofulous constitution. The white, transparent skin, pouting upper lip, tumid abdomen, bright red spot upon the cheek, dark and wiry hair, with the brilliant black or very light eye, are equally characteristic of the two diseases. When you find a case of this kind, in a child with a large head, and of precocious intelligence, you may set it down as one, in which caries of the spine is very likely to be hereafter developed. The same remark applies to diseases of the hip, white swellings, and scrofulous affections of the joints generally.

A child, then, of the character which I have described, runs about for two or three years, apparently, in the enjoyment of pretty robust health.—Suddenly he meets with some accident, as a fall, a blow, or the like. To this trivial cause, is usually attributed the long train of distressing symptoms which ensues—the constant proneness which has existed to the disorder, being kept out of mind or forgotten. In reality, the accident has merely ripened and brought into activity the latent seeds of disease, which have been long dormant. It cannot be termed a cause of that to which there has been so powerful a predisposition. In whatever part of the spine the caries may be situated, the early symptoms are usually alike. The child shows an indisposition to run about and join in the sports of his companions, but lays his little head in his mother's lap, complaining of the sensation of a cord across the region of the stomach. When seated, he draws his legs up under his chair; and he walks pigeon-toed, or with one toe overlapping the other: this subjects him to falls. He also speaks of uneasy sensations down his legs and thighs, which he draws up on his belly, when in bed. This sense of uneasiness is at last succeeded by actual paralysis. The power of motion is lost, or, if the child walk at all, he is constantly falling forward upon his hands. Generally, the lower limbs are affected, unless the caries exist in the upper dorsal and cervical vertebræ; but it usually occurs in the lower dorsal and lumbar vertebræ. This paralysis is not of the ordinary nature, as has been remarked by Pott. In common paralysis, you know, the power of sensation is lost, as well as that of motion, and the muscles are in a loose, flabby condition. Here it is otherwise: sensation remains, and the muscles are always in a state of more or less tenseness.—Thus the patient can hold his foot, although he is unable to place it. No doubt, in both cases, the paralysis arises from the same cause. In ordinary paralysis, it is pressure, occasioned by disease of the brain or nerves. In curved spine, this pressure is occasioned by destruction of the bodies of the vertebræ, or by a thickening of the sheath, enclosing the spinal marrow. If matter form, and travel extensively along the sheath, you have paralysis both of the upper and lower extremities. The muscles of the abdomen are usually much distended, from an accumulation of wind, consequent on a loss of nervous power. Combined with flatulence, there is considerable nausea; and the digestive functions are altogether disordered from the interference which the nerves of the stomach encounter. So that, after a short time, the patient suffers constitutionally as well as locally.

As the destruction of the bodies of the vertebræ proceeds, the spinous processes project, usually posteriorly.

When called to a patient, in whom you have reason to suspect the existence of caries of the spine, you should lay him on his face upon a bed, and run your fingers along the spine, examining with care each individual vertebræ. Press with your fingers upon them, one after the other, and when the patient cries out, you may conclude that you have found the seat of the disease. The number of the vertebræ affected is usually 2 or 4, and may be as many as 9 or 12; indeed, I have seen nearly the whole spine carious, with the spinous processes sticking out. I have a preparation in my cabinet of an individual, in whom the knees and chin were brought nearly into juxtaposition from this cause.

Some writers suppose that an actual dislocation takes place in spinal disease. This is not the case: At least no such thing as *primary* dislocation ever occurs, although secondary may be brought about. When the bodies of the vertebræ give way, displacement of this sort may ensue, giving rise to a very great degree of pressure upon the spinal marrow.

If you meet with a patient, before the disease has far advanced, you may do much anterior to the appearance of curvature and paralysis. Put him on a mattrass forthwith, and confine him steadily to the recumbent posture. The old writers recommend the use of machines, go-carts, and the like, to stretch the spine, as if it were dislocated. When you see modern surgeons resort to proceedings of this description, and employ machinery for the cure of *caries* of the spine, you may set him down as utterly ignorant of the pathology of the disease. He looks upon it as a displacement from softening, or a twist from over growth.

The vertebræ are not the only bones liable to caries. Other spongy bones are equally subject to it. The same plan of treatment is to be pursued for caries in every part of the body, whether it occurs in the spine, hip, knee, ankle, or wrist. The first indication, is to subdue the disposition which exists to the destruction of bony matter. This is not to be done, by an *operation* of any kind. You may remove a diseased bone, but, at the same time, you lay the foundation for disease in the adjoining bone. No man would attempt to cut out a diseased vertebra; why should he attempt to remove, in the same way, a carious astragalus, carpus, or sternum? The system is to be acted upon by internal remedies, and you are to keep the part affected perfectly at rest.

When genuine caries of the spine exists, a child will often make great efforts to hop about, but he will be finally obliged to desist, and go to bed. You will at once see the impropriety of disturbing a carious bone, when you reflect that any motion in the part, necessarily breaks up bony granulations as fast as they form. Dr. Physick told me that a gentleman once came to him, with a sore finger. which he had been troubled with for some time, and unable to cure. The Dr. said to him, "only keep your finger still, and it will soon heal." He accordingly placed a splint upon the finger, and it got well in a short time. So in chancre, motion of the parts and erections, are all that usually make them obstinate. If you could put a splint upon the penis, you would heal them up at once. There is no necessity for a splint in caries of the spine. The muscles there serve the purpose of splints. All that you have to do, is to keep off the pressure of the head and shoulders, by making the patient observe the recumbent posture. As matter is discharged, granulations are thrown out, forming bony splints, and the back becomes as strong as ever. The deformity, however, remains in the shape of an unnatural convexity or concavity, or lateral distortion. In addition to perfect rest, it was the plan of Dr. Physick to purge the patient pretty constantly, and form a drain by his bowels. For this purpose he usually kept the bowels open by a cathartic, once or twice a week. I myself have found caustic issues, on each side of the prominent vertebræ, very serviceable. They keep up constant counter-irritation, and discharge of matter; and under this course of treatment, I have seen the paralysis disappear. When this takes place, and not till then, I think machinery may be of use, *not to stretch*, but to give uniform support to the spine. It is impossible to prevent anchylosis, and any attempt of the kind must be most pernicious. No patient is fit to get off his back, until he has been lying on a mattrass for weeks or months. Never undertake to cure an individual affected with caries of the spine, until you have received a solemn promise, that your directions will be complied with. Let it be also understood, that he is to pass his fœces and urine, in the recumbent posture, and not leave his bed, upon any pretext whatever.

I present to you two examples of the disease. The one is still in progress; the other has been cured by anchylosis. This boy can give very little account of his case. All that he recollects is, that he has been in this state for two or three years; and he may probably remain so for two or three years longer. You observe that his case is complicated with hip disease. You notice the curvature from within outwards, and partially to the left side. His body is shortened to such a degree, that his hips approach his chest. His breast bone sticks out, like a turkey's. This boy would have been tall, but for this disease; he has lost some four or five inches of his height from the prominence in his back. The spinous processes stick out behind in a bunch, owing to the bodies of two, three, or four vertebræ having given way. You see these marks healed up, upon his back. He suffers no pain. There is a great difference in the duration of the disorder, in different individuals. Some are worn down, in a few months, by hectic, while others remain for several years in the same state, and finally get well with more or less deformity.

I show you to-day a collection of hunchbacks, from all parts of the house. Their backs, you see, are all sticking out, and they all say that, in early life, they met with some injury, to which they trace the disease, when the fact is, that 9 out of 10 owe it to a scrofulous constitution. This man here has had genuine caries, and not overgrowth. I will ask him what were the first symptoms of the disease, which he recollects. He replies, rheumatic pains: this corresponds exactly with the tenor of my previous remarks. He was cured, 20 years ago, with this solid bunch of bone upon his back, by the operation of nature—anchylosis. The man's full height would have been at least six feet: you see he is now a little fellow of five feet six. The ribs have dropped down to the hips, and there is more or less deformity of the whole chest. I should say, that, at least, 4 vertebræ had been involved in the caries.

Here is another case of spinal caries, with a curvature, involving the space of 7 or 8 vertebræ. You see how the shoulders are thrown back, and the breast projects. These patients have been all cured by anchylosis. I have already told you that to break up the deformity is to bring back the disease. You may therefore conceive the imprudence and ignorance of strolling bone-setters, who travel about the country, under pretence of curing broken backs, and straightening deformed limbs.

I now bring to your attention three examples of a different sort of spinal affection. These men are all rounded, in the back; but there are no spinous processes sticking out. This has not arisen from caries, but from rapid growth. The ligaments have become stretched, the patients have made exertions, the spine has given way, and this deformity has taken place. For these cases, a different plan of treatment would have been required. When a girl or boy grows rapidly, the mother puts on straps to support the back; the consequence is, that the muscles do not act, they give no support to the bones, and down they go. The bones, you know, act as support to the muscles, and reciprocally the muscles to the bones. It is the height of impropriety, in such instances, to reduce the child to the recumbent posture. What then is the proper course to pursue? To increase the action of the muscles by free exercise in the open air; if you have a girl to deal with, prohibit corsets, and clothe her loosely; encourage the use of dumb-bells, 5 or 6 times a day, and make the child hop, skip, and jump about, with all the zeal imaginable. If these men had been so treated; if, when children, they had been made to play about, and had had the benefit of gymnastic exercise, their backs would have been now straight. One of these men tells you he has been a shoemaker from the age of 11 years: it is no wonder, then, he should have been, with his predisposition to disease, doubled up into a knot, as you see him. Of course, now, there is no chance of a cure; the bones are fused into a solid mass. They should have been prevented from getting into this state of deformity, by exercise, and all the other means pointed out.

Dr. Jackson followed Dr. Gibson.

Gentlemen: Dropsy may be defined as consisting in serous effusions into the great cavities, and cellular membrane of the body. Our remarks at this time will be confined to the General Pathology, and the Therapeutics of Dropsy. The special forms will be treated of as cases occur in the wards. This pathological state may happen under a variety of conditions.

Dropsy is rarely idiopathic; it is consecutive on other lesions. One patient presented to you the other day, had anasarca, with commencing ascites, but on close investigation, the second sound of the heart was found unnatural, indicating an affection of the semilunar valves. Yet in another patient in whom too the same sound existed, there were no dropsical effusions. Another patient had dropsical effusions into the cellular tissue of the lower extremities, and into the abdomen, diminishing in both situations. His urine coagulated by heat, and by nitric acid, pointing out disease of the kidneys. Other cases before you exhibit effusions into the chest, the consequences of pleuritic inflammation; whilst cases of pleurisy are met with, unattended with a similar result. Dropsy is also the last link in the chain of the morbid degenerations of the organ, it is the closing act of the pathological drama, when the whole organism is in ruins. You had a case of this kind before you a few days since. The patient, who had led an intemperate life, was labouring under ascites unattended with anasarca. It was an instance of simple ascites; but his health had been for a long time declining, and his constitution was undermined. He was in the last stage of emaciation, and his face had assumed the hippocratic aspect—*facies Hippocratica*, so named from the graphic description that writer first gave of it.

From the numerous dissections made in this house of similar cases, no doubt the liver and the whole digestive apparatus of that patient were changed in their structure, and their identity lost.

It is thus evident that in dropsical effusions there are two elements, the one some especial affection of a tissue or organ; the other a modification of the blood,—that is the proportion of water in the blood is excessive, whilst the solid and plastic elements are in much less quantity. From this circumstance, the same vascular reaction proceeding from the same causes, will produce serous effusions —forming dropsy in individuals whose blood is in this state; while in others differently constituted, lymph will be thrown out, false membranes, or other new formations will take place, or abscesses be formed.

Dropsical effusions may happen at all periods of life; from the fœtus in utero, to the extremest old age. It was only a few days ago that I was consulted by a gentleman regarding a lady who in her three last pregnancies, aborted in her sixth month; the fœtus each time becoming affected at that period with dropsical effusions.

Some persons are more liable to this form of disease than others; those of the lymphatic temperament are especially subject to dropsy. Such persons have much less red blood than those of the sanguine habit of body, with a greater proportion of serum; hence the tendency to effusion. The nervous and sanguine temperaments are very rarely affected, unless the temperament becomes changed and altered from the effects of disease.

It is necessary to discriminate between acute inflammation of the serous membranes, and dropsical effusions. This is not always an easy task. When inflammation attacks a serous membrane, some degree of effusion always takes place. But in well constituted individuals, and those of sanguine temperaments, the proportion of serous fluid is small; lymph is thrown out, in membraniform layers, or is mixed with the serous fluid, rendering it lactescent or turbid in appearance. In others the proportion of serous fluid is more considerable; still it is mixed largely with albuminous exudations, whilst false membranes cover the serous linings of the cavities, or the serous membranes are entirely changed in structure. While the serous membranes, and the effused fluid exhibit these appearances, the disease is a serous inflammation—a pleuritis—a peritonitis—an aranchitis

—a pericarditis, and not a dropsical affection. We meet with cases, however, in which the inflammation having passed into the chronic stage, or having terminated, leaving the serous membranes more or less changed in structure, and affected in function, the effusion continues to augment, and a genuine dropsy thus ensues, as a consequence of the inflammatory disease.

From the evidence of these facts it appears to me that we must look for the cause of dropsy in the condition of the blood; and also in the different results of inflammatory action in the serous membranes of different individuals.

Having given you this rapid sketch of the view I take of the nature of dropsies, I will now pass to the consideration of the different forms under which the disease is manifested; and first I will bring to your notice *inflammatory dropsy*. Some physicians are of opinion that dropsies are always inflammatory; this was the opinion of the late Dr. Rush, it is the doctrine of Ayre and is advocated by many. That inflammatory dropsy exists, there cannot be the least doubt, but the cases are uncommon. They are usually produced by exposure to cold, or damp—a sudden check of perspiration, taking very cold drinks when heated, and similar causes. I recall one instance which was in the house several years ago. The weather suddenly became cold, and in one night the river was frozen over; vessels which were to have sailed the next day, were thus detained, and persons were employed to cut a passage in the channel. Now this man was exposed for a number of hours to the cold in a boat half full of water. In forty-eight hours he was attacked with general dropsy. He had anasarca of the lower extremities, the legs were enormously swollen; the scrotum and abdomen were distended with the fluid. He had in the first place been attacked with pains; he had some cough, and a frequent tense pulse. He was bled, and actively purged; the symptoms of inflammation reduced down, and in three weeks he was discharged, cured. A similar case happened at the same time; this patient was a drunkard; being intoxicated he was all night exposed to the cold, and next day had ascites. He was similarly treated and cured in the same space of time. The late Professor Wistar was accustomed to relate a case that fell under his observation, which I take to have been of the same character. The Professor used to quote it as an instance of the formation of dropsy in a manner too rapid to be explained, by the ordinary doctrines prevailing at that time. A young lad was skaiting, and while heated, and in a profuse sweat from his exertions, laid down on the ice. He was soon seized with a chill; taken with pains in his limbs and body. The next morning the abdomen was largely distended with serous effusion, a complete anasarca had formed.

Now these cases I regard as instances of simple inflammatory dropsy. Now, you must understand that *inflammatory* dropsy is not the same, or to be confounded with acute inflammation of some one of the serous membranes, accompanied, as has already been shown, with effusions of sero-albuminous fluids. There is a strong, well marked tendency in these membranes to terminate, when attacked with inflammation, in effusion into the cavities which they line; the effusion in this instance is the consequence—is consecutive to the inflammation. It is sometimes very difficult to discriminate, whether you have hydrothorax or pleuritis, arachnitis or hydrocephalus.

Inflammatory dropsy may be known first, from the cause which produces it, which is generally exposure to cold. Secondly, from the suddenness which it forms after the action of the exciting cause: the dropsical effusion appearing ab initio. Thirdly, it is usually attended with pains having resemblance to rheumatism; cough and pain in the breast are also present, it is accompanied with febrile action, or the pulse is frequent and tense. Many of the symptoms resemble a common cold. The blood drawn presents a buffed coat.

This patient now before you, is a specimen of the disease of a mixed character, or of inflammations terminating in dropsy. This man was in the house some time previous, and he then had dropsical effusion, he got better and went out. A short time afterwards he was attacked with pain in the left side of the chest; dropsical effusions again appeared, and he re-entered the hospital. It is stated, (for I have not before seen the case,) that the dull sound which you find existing throughout the left side of the chest, was at first only partial, and that it gradually extended, until as now, it occupied every portion, from the clavicle to the base of the chest, anteriorly as well as posteriorly. Now, this dead, dull sound, proceeds in this case from effusion into the pleura. We have the evidence of this, first, from the gradual increase of the sound; secondly, from its varying, (as Dr. Pennock, who attended the case, has just mentioned,) with the position of the patient; thirdly, from the sign of the former dropsical effusions; and finally, from the other dropsical effusions into the abdomen and extremities. You, observe also, that the resonance of the right side of the chest preserves its natural character. Respiration is also natural, rather loud in the right lung, while it is absent in the left. It is then the left pleura in which the effusion exists. From this circumstance—the freedom of the right pleura and lung from disease—the patient, as you observe, can keep the recumbent position, and does not present that distressing state of breathing, generally mentioned by writers, as a symptom of hydrothorax. You notice further, when I make firm pressure on the intercostal spaces of the left side, the patient expresses a feeling of suffering—it gives him acute pain. This to me is an indication of chronic inflammation, existing in the pleura; its sensibility is unnaturally augmented. Chronic pleurisy is often latent, and is not expressed by the rational signs; pressure in this way will often manifest the morbid sensibility,—one of the signs of pleuritic inflammation. This circumstance, taken in connection with the previous pain in the left side; gives a strong presumption, that we have chronic inflammation of the pleura—with or without tubercular development.

We have in this patient not only hydrothorax of the left side, but dropsical effusion has also occurred into the abdomen. The abdomen here is very tense; a very slight percussion produces the wave, or shock, transmitted from one side of the abdomen to the other. Here then is evidence of effusion into the cavity of the peritoneum. The heat of the abdominal integuments, I find to be above that of the rest of the body. I am disposed to believe, from that fact, that peritoneal inflammation of a chronic form is present. Now it is very common for membranes of the same class to take on the same morbid action; and I have but little doubt but that the serous lining of the abdomen is affected similarly to the pleura. In this case there has existed strong evidence of inflammation, and should a chance for examination occur, we shall probably have in the peritoneum signs of inflammatory action. In cases of an inflammatory nature the antiphlogistic means are to be employed. You bleed generally, or locally, according to the indications; you purge, and give diuretics. The best purgatives are those called hydragogues. A very good combination to form a hydragogue cathartic, is cream of tartar, jalap and gamboge—it is probably one of the best. But there are instances where you must be cautious in the employment of blood-letting. You must reduce down inflammation, but at the same time you must be careful not to produce further effusion; for, by depriving the blood of its crassamentum, you increase the tendency to effusion. When bleeding is not admissable, you employ then those remedies which are calculated to throw off the aqueous portion of the blood, having in mind at the same time, to improve the nutrition of the patient. The food should be of *good quality*, but bland in character. Weak chocolate, milk—if it suit the digestion of the patient—and cream. The farinaceous vegetables are to be selected. Good strong concentrated broths, the *consommés*, are often excellent. Weak broths will not answer, they do not furnish sufficient nutriment, they do not digest well, and inundate the stomach. Boil the soups into a jelly, and give of these a teacup full or two at a time; light meats may also be allowed the patient.

In inflammatory dropsy, advantage is often derived from a slight mercurial course. It should not however, be pushed beyond a very gentle ptyalism.

After the inflammatory action has been subdued you may also use chalybeates; these may sometimes with advantage be combined with the hydragogue cathartics, as carbonate of iron, cream of tartar, jalap and powdered gentian.

Dropical effusions often follow scarlet fever; in 4 or 5 cases out of 12, they are the sequelæ of the disease. There is no necessary connection between their intensity and that of the primary affection. Cases of the disease, so slight as to require no particular treatment, will often be followed by dangerous effusions. Dropsy succeeding to scarlatina is of an inflammatory grade. It is often preceded by pains in the joints and limbs. The pulse is frequent and tense, and the blood drawn is buffed and cupped.

Now you will find a small bleeding often predisposes to a more favorable condition—renders the operation of remedies more certain, and arrests the tendency to effusion.

The effusion after scarlatina may occur every where except in the arachnoid. I have never seen or heard of its taking place in that serous tissue. It often occurs very suddenly and with great violence, where the disease in itself has been very light. A boy, in a family where I have been in the habit of attending, had passed through an attack of scarlatina, but it was so light that I was not even consulted. He was going about again, when, all of a sudden, he became anasarcous; in the night he was attacked with dyspnœa which threatened suffocation; the resonance of the chest was rapidly diminishing; he was constantly crying out, "I shall die"—"I shall suffocate." After abstracting some blood, I ordered him colchicum, elaterium, with sweet spirits of nitre; as soon as these produced several copious watery evacuations, he was relieved. In dropsy following scarlatina I know of nothing which controls the disease so well as digitalis. In this form of dropsy I place great reliance on this remedy; it seldom fails to prove decidedly useful. I push it constantly for the first 24 or 48 hours, watching carefully its effects. In dropsy depending upon disease of the heart, digitalis is of signal utility. In simple ascites I have never known it to be of the slightest advantage; I have reduced down the pulse as low as thirty in minute, without any benefit whatever resulting.

The most intractable form of dropsy is that depending on lesions of the heart, or obstructions to the circulation from obliteration of the veins.—These cases never are cured. You may get rid of the effusion, but it speedily returns. In hypertrophy with dilatation, dropsical effusions do not take place until the circulation has become very embarrassing, and the functions of nutrition impaired.—Previous to this the disease may be dissipated. Digitalis in these cases is a precious remedy, and often produces relief in the most prompt manner. It frequently too procures the temporary dispersion of the effusion, when the affection has become incurable. In dropsy depending upon simple dilatation of the heart, digitalis and debilitants are doubtful remedies; general nutrition is impaired; there is a deficiency in the nutrition of the organ; it loses the powers of action and resistance; the circulation is more enfeebled, and the dilatation increases. The indication then is to combine with hydragogues or diuretics the mild tonics, and employ a nourishing diet.

There are other forms of dropsy less frequent; such as those depending on the obliteration of the larger veins. It is then a very incurable disease. In this country we have no records of such cases; but in Europe, where antopsies are more frequently and more carefully made than generally with us, there are many cases reported.

Dropsy is very often consecutive to remittent and intermittent fevers. Our wards during the great epidemic, continuing from 1820 to 1826, were continually filled with such cases. Every winter during that period we had every form of dropsy bearing that lineage. Since those diseases have disappeared, and given way to another type of disease, we have comparatively few instances of the affection. When intermittents are prevalent you will frequently meet with cases of dropsical effusions occurring before the cessation of the intermittent. In those cases I succeeded by combining with the bark—administered for the intermittent—the alkalies and cream of tartar. In dropsies eventuating on fevers of this character, the chalybeates are very efficacious. With mild purgatives they are the most effective means in reducing enlargements of the spleen and liver. The phosphate of iron may be employed, but I am partial to the tartrate of iron dissolved in a simple infusion of gentian, or infusion of gentian and rhubarb.

Remedies which combine in themselves several properties, are very useful in the treatment of dropsy. The *kahinka* has lately obtained a great reputation in Europe in the treatment of this disease. It is the root of a plant, a native of Brazil. It is hydragogue, tonic, stimulant, and diaphoretic. The infusion or the extract are the usual forms in which it is administered. As popular remedies in the disease several articles have obtained a great reputation; they are all of the above description. *Eupatorium* is one of them. Given in hot decoction it proves emetic, purgative and diaphoretic; at the same time it is endowed with tonic virtues. The *spartium* or *broom tops*, which are diuretic and cathartic, have also been recommended, and the *Indian hemp—apocynam canabinam*—has proved successful in a few cases, it acting as an emetic, cathartic, and diuretic. Tobacco in the form of tincture has been used with some advantage.|

Amongst the remedies for dropsy, *nitrate of potassa* has obtained some reputation. It is decidedly diuretic in its operation, but in no instance have I obtained any well-marked beneficial results from it in dropsical effusions. It often deranges the digestive functions and does harm.

The drinks for patients laboring under dropsical effusions should generally be rendered diuretic in their properties. This can be effected by making them of various infusions—as the Juniper berries, or adding to them sweet spirits of nitre, or cream of tartar. Dr. Parrish of this city, a very successful practitioner, frequently directs one made of an infusion of horse-radish, ginger, mustard seeds, and Juniper berries, in cider. I have employed it with good effects. In cases of intemperate persons, who must be allowed some stimulus, gin may be permitted, mixed in moderate portions in their drinks.

There is another form of dropsy depending upon anemia and chlorosis. In these affections the solid elements of the blood are greatly diminished. The proportion of crassamentum to serum—in health 14 to 10—is reduced to 6 or 7 to 10; in some cases even less. The aqueous element is unduly increased, and a tendency to general dropsical effusions result. We have here great alteration in the constitution of the blood. In these cases we will find a mild tonic and stimulant treatment, with the most nutritive diet, the proper mode to be pursued. The martial preparations are here of wonderful efficacy. Treated in an appropriate manner these cases generally recover.

There is one form more of dropsy, depending upon disorganization of the kidneys, that it will be necessary to notice. Bright was the first person who called the attention of practitioners to this affection. It is often designated by *Bright's disease.* The distinctive character of this order of dropsy is the constant presence of albumen in the urine. If you take the urine of a person laboring under this disease, place it in a spoon and hold it over a candle, you will find it coagulates readily. In very bad cases it becomes a solid, hard mass. These patients complain of pain in the back; have a pulse frequent and tense, with scanty urine. I do not think that the presence of albumen in the urine is always evidence of organic disease in the kidneys. Blackall mentions it as occurring in the inflammatory types of dropsy. I have known it happen in the dropsy consecutive to scarlatina, as there could be no disorganization of the kidneys, as the patient recovered. This form of dropsy must be treated with reference to the organic affection. Cups and blisters are to be directed to the loins; permanent issues and blisters to the loins may be necessary.—The internal remedies found most useful are some of the diuretics. Cream of tartar in weak solution is to be administered. Infusions of the tonic diuretics in *uva ursi*, &c., in which is dissolved bi carbonate of potassa, or soda, is of great utility.—Bright speaks highly of a combination of *cicuta* and *squill.* When the organic alteration is very considerable, no permanent advantage is to be derived from any course that may be adopted.

I have now presented you with a rapid outline of the general pathology of this disease, with the various modifications it presents, together with the numerous methods that may be employed to combat the effusion. When these means fail to relieve, and the patient suffers from the distention of the fluid, we often resort to mechanical means, by which the water may be removed from the cavities. In hydrothorax the operation of *paracentesis thoracis* is sometimes performed. You are all acquainted with the mode of this operation.

In the abdomen tapping is frequently resorted to; but, from its uniformly fatal results, both in this establishment and in private practice, I have been disposed to abandon it. In one half of the cases, in which it has been performed, death has ensued in a few days, from the development of acute peritonitis. This constant, almost invariably fatality, I am disposed to attribute to the existence of chronic peritoneal inflammation, in a large portion of the cases of ascites. When it results from cardiac disease the operation is seldom performed. There is nothing perhaps more difficult to detect than chronic peritonitis. In one case which we examined of a patient who had died after the operation of *paracentesis abdominis*, we found the whole peritoneal surface covered by coagulable lymph, and the convolutions of the intestines agglutinated into a solid mass.

There are some cases though in which tapping has been successful. There is a lady in this city, whose case has been mentioned to me, who, for the last ten or twelve years, has been regularly tapped twice or three times a year, and each time several gallons drawn off. The belly becomes distended and uncomfortable, she is then tapped and relieved. The nature of the case I do not know, but she is a woman of active habits of life. Van Swieten mentions a number of cases of a similar character, from which enormous quantities of water were taken at various times. Before concluding this subject, I may mention several very extraordinary cases, and singular terminations of the disease, which have occurred to me in the practice of this establishment. In a case of ascites the whole of the water in the abdomen was evacuated by spontaneous vomiting in the space of 24 hours; the patient died immediately afterwards, exhausted down. How this took place, whether by endosmose through the coats of the stomach, or by absorption and exhalation, it is impossible to know. In the case of a colored girl the whole fluid was evacuated through the vagina in the course of a few hours; as in the former case the patient died immediately afterwards. In another case I applied a blister to the abdomen; a copious serous discharge continued for several days, and the ascites disappeared. I was induced to try the same remedy in another instance, but with different results. The patient died, and the skin of the abdomen and of the outside of the left thigh was found dissected loose by a sero-purulent effusion into the cellular tissue. During life no symptoms indicated the existence of this inflammation. The most extraordinary case I ever met with was one in which I resorted to accupuncturation. This was a woman laboring under general dropsy, hydrothorax, ascites, and anasarca. She was given up by all the physicians in the house, and was considered as in *articulo mortis;* indeed, her body had already been bespoken by one of our practical anatomists. A number of punctures were made in her lower extremities, and the whole of the water was evacuated. The punctures discharged so freely that the water soaked through the bed, and was caught in a bucket beneath it. In the course of a week or ten days she was free from this general effusion. She entirely recovered, became an assistant nurse in one of the wards, and died 18 months afterwards of some acute affection. Accupuncturation is to be preferred in relieving the distention of the extremities to scarification, which was formerly employed—it is a safer practice.—Some physicians have gone so far as to assert that the needle is always a safe means of evacuating the effusion from the extremities, penis or scrotum, when too much distended; and that you need never anticipate any evil effects from its use. My own experience does not correspond with this. In a case in which I employed it, sphacelation was produced in the whole limb; but the patient was in such a state that he could not have survived but a few days, the organic lesion was hyperthrophy with dilatation of the heart, with embarrassed circulation. If you employ occupuncturation, and have any doubts as to the power of the skin to support it, you had better insert your needles high up in the limb, where the vitality is greater. There will be less risk of mortification.

